# What parameters really matter? Linking exposure and effect models to identify sensitive parameters for model-based aquatic risk predictions of pesticides

**DOI:** 10.3389/ftox.2026.1984119

**Published:** 2026-10-27

**Authors:** Paula Scharlach, Dimitrios Skodras, Andreas Focks

**Affiliations:** 1 Institute of Mathematics and Institute of Environmental Systems Research, Osnabrück University, Osnabrück, Germany; 2 Department Modelling and Bioinformatics, Fraunhofer Institute for Molecular Biology and Applied Ecology IME, Schmallenberg, Germany

**Keywords:** aquatic toxicity, environmental risk assessment, pesticide fate model, TKTD modeling, sensitivity analysis

## Abstract

Environmental risk assessment (ERA) of pesticides is essential for protecting non-target organisms and supporting sustainable agricultural practices. This study links exposure and effect models for the prediction of pesticide risk and performs a global sensitivity analysis to identify the most influential parameters for maximum concentrations and risk indices. We coupled the FOCUS surface water Step 3 model (TOXSWA combined with PRZM) with the General Unified Threshold model of Survival (GUTS) to translate time-resolved exposure profiles into lethal profiles (LP_50_) values, capturing time-dependent toxicity and recovery processes. This approach offers a dynamic and mechanistically-based quantification of risk compared to traditional static risk quotients, which are based on single time-point concentrations. After prescreening 20 model parameters using a Morris sensitivity analysis, we selected the sorption coefficient and water degradation time as substance-specific properties, water depth as a key water-body parameter, and catchment size to capture the influence of loadings from agricultural practices for further in-depth analyses. Sobol sensitivity analyses of these four parameters were performed to assess the global sensitivity to simultaneous changes in these parameters. We found that while the maximum predicted environmental concentrations (PEC_max_) are primarily driven by water depth and catchment size, risk quantified by LP_50_ values is most sensitive to catchment size and so to loadings. Furthermore, degradation time does not influence PEC_max_, but its sensitivity increased for LP_50_ values associated with slower kinetics. These findings show that parameters to which peak exposure is most sensitive are not necessarily the same as those to which a time-resolved risk metric is most sensitive. Whether this added mechanistic detail improves the prediction of effects in the field remains to be tested by benchmarking against monitoring data and simpler metrics.

## Graphical Abstract

**Figure:**
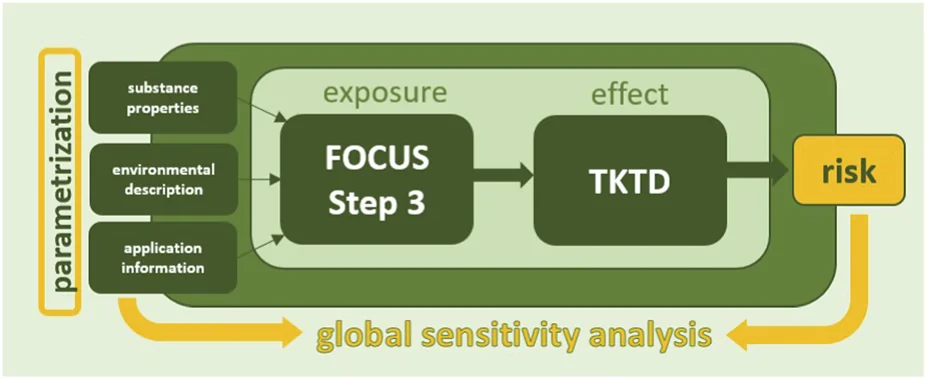

## 1 Introduction

Pesticides are widely used in agriculture to maintain crop productivity (Tudi et al., 2021), but their EU authorization requires comprehensive environmental risk assessments (ERAs) to safeguard human health and environmental integrity (Regulation EC No 1107/2009). To manage the high cost and time requirements, ERAs typically employ standardized frameworks that combine laboratory testing with modeling following a tiered approach from simple screening-level predictions to increasingly complex models (EFSA, 2013; Ozoh et al., 2026). However, as models increase in complexity, so does their demand for detailed input data, including substance-specific properties (e.g., water solubility, degradation rates, sorption coefficients), site-specific environmental information (e.g., temperature, pH, slope), and detailed exposure scenarios (e.g., application doses and times). While large amounts of relevant data already exist, they are often fragmented across diverse databases, limiting their integration into ERA models and reducing overall efficiency (Axelman et al., 2024; Ozoh et al., 2026). In practice, this fragmentation forces a choice between costly, potentially unnecessary experimental data collection and the use of uncertain default values, which often delays regulatory decision-making (Axelman et al., 2024).

This challenge is particularly evident when coupling higher-tier exposure models with effect models to refine risk predictions. In the regulatory framework provided by the FOrum for the Co-ordination of pesticide fate models and their Use (FOCUS), Step 3 and 4 employ time-dynamic differential equations across ten representative European scenarios to calculate predicted environmental concentrations (PECs) (FOCUS, 2001). While these provide a high level of detail, it has been demonstrated that PECs are not always conservative and may underestimate actual concentrations in surface waters (Knäbel et al., 2012, Knäbel et al., 2014; Scharlach et al., 2026), highlighting the necessity to verify the parameter assumptions made in the models. Traditionally, ERA relies on comparing model-based PECs with effect thresholds (Rico et al., 2021; Schmolke et al., 2010), but the European Food Safety Authority (EFSA) also endorsed semi-mechanistic toxicokinetic-toxicodynamic (TKTD) model approaches as a ready-to-use method for effect assessment (EFSA et al., 2018). Specifically, the General Unified Threshold model of Survival (GUTS) provides a robust framework for estimating time-resolved risk metrics, such as lethal profiles (LP_50_), which can be coupled with FOCUS Step 3 outputs to quantify risk (Ashauer et al., 2013; EFSA et al., 2018).

The coupling of FOCUS Step 3 and GUTS represents a powerful tool for risk quantification, but it also creates a high-dimensional parameter space. The combined complexity of exposure-related variables (e.g., hydrology, degradation, partitioning between environmental compartments) and effect-related parameters (e.g., quantifying the TKTD processes) raises a key question: which of these inputs actually drive the predicted risk?

In this study, we address this question by applying a global sensitivity analysis (GSA) via Morris prescreening (Morris, 1991; Paleari et al., 2021) and subsequent Sobol’s variance-based decomposition (Sobol, 1990; Sobol, 2001) to the coupled FOCUS Step 3 and GUTS models. By quantifying the influence of parameters on the maximum PEC (PEC_max_) and the risk quantifier LP_50_, independently of specific pesticides or locations, this study identifies sensitive parameters to streamline data requirements and prioritize the measurement of critical parameters. However, the models may be highly sensitive to a particular input parameter, even if that parameter remains relatively stable in the real world. This means high sensitivity does not necessarily correspond to high ecological uncertainty. The discussion of the results addresses actual environmental variability and how it is represented in the models. Nevertheless, identifying sensitive parameters improves the efficiency and robustness of regulatory ERA and supports a transition toward a simplified, systems-based ERA (Duquesne et al., 2026).

## 2 Materials and methods

To investigate how different parameters and their interactions affect the model results, we performed a GSA. We first pre-screened a set of 20 parameters describing substance-specific properties, water-body and soil characteristics using the Morris method. From this, a subset of four most relevant parameters was identified based on their total sensitivity and interaction potential, which were then analyzed using the variance-based Sobol method with Saltelli’s quasi-random sampling (Saltelli et al., 2010). Finally, we used total-order Sobol indices to establish sensitivity hierarchies for varying sorption coefficients and discussed parameter variability in reality. This three-stage framework, a Morris pre-screening, followed by a full Sobol analysis and the comparison of sensitivity hierarchies, ensures a comprehensive evaluation of parameter influence while maintaining computational efficiency.

### 2.1 Linking exposure and effect models

In this study, we combined two established modeling approaches to predict time-resolved risk for non-target organisms. First, we used the FOCUS surface water Step 3 model to generate time-resolved exposure concentration patterns, specifically PECs. The fate of the pesticide in the soil is calculated with the Pesticide Root Zone Model (PRZM), considering degradation, adsorption to organic carbon in the soil (Koc) and volatilization (Carsel et al., 1985). The main transport process covered here is runoff, a transport over the soil surface. Drainage, leaching through the soil, was not included in our PEC calculation. Simulated pesticide loadings from the catchment area are used in Toxicity Surface Water Analysis (TOXSWA) to simulate concentrations in the water column and sediment, accounting for dilution, sediment interactions, and degradation (FOCUS, 2001). In this study, we focused on water concentrations, which served as input for the GUTS model to predict ecological effects on (non-target) organisms.

Although the environmental conditions in our model were primarily based on the original FOCUS R1 pond scenario and winter cereals (see FOCUS, 2001), we expanded the scope to cover additional scenario properties by systematically varying selected parameters. The R1 standard assumptions are representative of German and Central European environmental conditions, and we held the following general agro-environmental characteristics constant: temperature, recharge, precipitation, slope, soil drainage, water body type and crop management. Our sensitivity analysis instead focused on parameters describing soil and water-body characteristics, as well as substance-specific properties that influence environmental transport processes.

To predict environmental effects on organisms, we linked results from the FOCUS Step 3 model to the GUTS models. While TOXSWA provides hourly PEC values for 1 year, we extracted the maximum daily concentrations to create daily-resolved time series as input for the GUTS models (Jager and Ashauer, 2018). This approach was adopted to optimize computational efficiency when calculating risk predictions and to prioritize parameter sensitivity over specific absolute risk values. These daily-resolved PECs were then used in the GUTS models to translate external concentrations into damage dynamics and, finally, to predict survival probabilities. Risk was quantified using LP_50_ values, which can be interpreted as multiplication factors that would reduce the survival probability to exactly 50% (EFSA et al., 2018). We used only the stochastic death (SD) model variant of GUTS, in which all individuals have the same effect threshold *m* and once internal concentration exceeds this threshold, the probability of dying increases proportionally based on the killing rate *b* (see SI, section A; Jager et al., 2011). However, we considered three different types of kinetics, fast, medium and slow, by defining the parameters accordingly. Based on a generic substance with the parameter set: *k*_*d*_ = 0.3 days^-1^, *b* = 0.5 L/(moll · d), and *m* = 2.5 mol/L (EFSA et al., 2018, Fig. 8), we accelerate kinetic processes by increasing the dominant rate constant to *k*_*d*_ = 10 d^−1^ and simultaneously reducing the threshold value to *m* = 1 mol/L (Table 1). By increasing *k*_*d*_, damage increases more rapidly with increasing exposure concentration, which can result in faster death mechanisms. With a lower threshold value, hazard begins earlier, and even low damage values can lead to mortality. In contrast, reducing *k*_*d*_ to 0.1 d^-1^ leads to more buffered peaks, resulting in lower mortality. Together with an increased threshold value of *m* = 10 mol/L, the hazard will occur later, and organisms will survive longer, representing slow kinetics. Background mortality *h*_*b*_ was assumed to be zero for all model runs that focus solely on pesticide-induced mortality.

**TABLE 1:** Generic parameter sets for the GUTS-RED-SD model. The kinetics of the GUTS-RED-SD model was defined as fast, medium or slow by adapting the dominant rate constant *k*_*d*_ and the threshold value *m*. The background mortality rate *h*_*b*_ and killing rate *b* are fixed for all models.

Kinetic	*h*_*b*_ [1/d]	*k*_*d*_ [1/d]	*b* [L/(mol · d)]	*m* [mol/L]
Fast	0	10	0.5	1
Medium	0	0.3	0.5	2.5
Slow	0	0.1	0.5	10

All model calculations were performed in Python (version 3.14.2). For the PEC calculations, we used the existing PRZM and TOXSWA software and called the models via Python scripts. GUTS applications were performed using the Python package *mempyGUTS*, which is publicly available on GitLab (https://gitlab.uni-osnabrueck.de/memuos/mempyguts). As this is theoretical work and based on parameter variations that do not represent real substances, no specific aquatic species was targeted. Instead, the study aims to identify generic key risk drivers from a selected set of parameters.

### 2.2 Parameter selection and value ranges

To evaluate the sensitivity of PEC_max_ and LP_50_ values, we systematically varied the input parameters for the PRZM and TOXSWA models. The initial screening focused on 20 parameters selected based on their relevance for the aquatic environment: hydrological parameters (characterizing pesticide input from catchment areas and water dilution), water body parameters (defining pond geometry) and sediment parameters (describing sediment properties). Furthermore, we included substance and transport parameters specific to runoff in the PRZM model and shortened the permitted value ranges to realistic conditions (see Table 2). For instance, we limited the height of the weir crest in the pond to the range 0.2–2 m, which can be interpreted as the actual water depth. FOCUS assumed an average water depth of 1 m (FOCUS, 2001), and Wachholz et al. (2026) determined a median water depth of 1.5 m in Germany. Also, Zębek and Szymańska (2017) reported a maximum water depth of 1.5 m in Polish ponds. Nevertheless, water depths can vary widely, and in Germany, values up to 170 m have been recorded (Wachholz et al., 2026). However, because the FOCUS scenarios tend to focus on small water bodies and worst-case assumptions, we consider a depth of up to 2 m realistic. Another example is the size of the surrounding area from which excess water flows into the pond. We refer to this parameter as ‘catchment size’, which is defined for FOCUS ponds as 0.45 ha. In general, a weak positive relationship was found between water body size and catchment size, but not all small ponds have small catchment sizes (Novikmec et al., 2016). The proportion of intensive land use in a catchment influences the pesticide contamination in adjacent water bodies, and Davies et al. (2008) determined 18 ha as an average catchment area, whereas Zębek and Szymańska (2017) determined a maximum size of 1.5 ha. FOCUS stream scenarios assume a catchment area of 100 ha with only 2% treated with pesticides (FOCUS, 2001). As an average of these very diverse values from the literature, we decided to include sizes between 0.1 and 10 ha in our sensitivity analysis. Degradation times (DT_50_) in water are used as a measure of persistence in aquatic risk assessment and vary significantly due to the processes involved, e.g., photolysis, hydrolysis, oxidation, and biological degradation. They are difficult to measure in the lab but are nevertheless important substance parameters (Pereira et al., 2016). To cover a broad range of pesticides, we set the degradation time at a minimum of 0.1 and a maximum of 100 days. Additionally, the sorption behaviour (Koc) ranges from 10 to 10000 in our analyses. This parameter plays an important role in ERA as it describes the mobility potential of the pesticide from the field to water and is therefore one of the key parameters in risk predictions (Pereira et al., 2016). For additional values, like water body length, width and the slope of the water system, we compared FOCUS assumptions and scientific literature on monitoring studies. It should be noted that the results of the sensitivity analysis depend on the defined parameter spaces, and our selection does not cover every possible parameter combination in reality.

**TABLE 2:** Parameters included in Morris prescreening to identify sensitive parameters. The original names from the TOXSWA calculations were changed to improve clarity and enable quicker identification. Furthermore, the parameters are categorized according to their function in the model. The value ranges describe all parameters considered in the global sensitivity analysis.

Parameter in FOCUS	Parameter name	Parameter description	Category	Value range
HgtCrePnd	water_depth	Height of the weir crest into pond	Hydrology	[0.2, 2.0] m
WidCrePnd	water_width	Width of the weir crest into pond	Hydrology	[0.2, 2.0] m
AreaSurPndInp	catchment_size	Size of surrounding area discharging excess water into the pond	Hydrology	[0.1, 10] ha
QBasPndInp	inflow_pond	Base flow, i.e. inflow into pond	Hydrology	[0.1, 50] m^3^/d
Len	wb_length	Water body length	Water body	[30, 100] m
WidWatSys	wb_width	Width of the bottom of the water system	Water body	[30, 100] m
SloSidWatSys	side_slope	Side slope of the water system	Water body	[0, 10] m
ConSus	susp_solid	Concentration of suspended solids	Water body	[0, 100000] g/m^3^
CntOmSusSol	mass_ratio_oc	Mass ratio of organic matter in suspended solids	Water body	[0,1] g/g
Rho	bd	Bulk density	Sediment	[500, 20000] kg/m^3^
CntOm	Om	Organic matter mass content	Sediment	[0.005, 0.3] kg/kg
ThetaSat	theta_sat	Saturated water content	Sediment	[0.5, 0.8] m^3^/m^3^
MolMas	mol_mas	Molar mass of substance	Substance	[100,700] g/mol
SlbWatRef	wat_sol	Water solubility of substance	Substance	[0.1, 10000] mg/L
PreVapRef	vap_pres	Saturated vapour pressure of substance	Substance	[0.000001, 0.1] Pa
DT50WatRef	dt50_wat	Half-life in water	Substance	[0.1, 100] d
DT50SedRef	dt50_sed	Half-life in sediment	Substance	[1, 100] d
DT50	dt50_soil	Half-life in soil (only relevant for PRZM, k_dis)	Substance	[0.1, 365] d
KomSed	Koc	Freundlich coefficient of equilibrium sorption for sediment (Kom = Koc/1.724)	Substance	[10, 10000] L/kg
Application time	app_date	Application date (based on suggestion by AppDate for winter cereals in R1 scenario, considering emergence)	Application	[316, 346]

### 2.3 Prescreening with Morris

Given the high dimensionality of the model pipeline, we used the Morris method to identify the most sensitive parameters for risk predictions. The Morris approach is a GSA with a computational cost comparable to that of a local sensitivity analysis, making it an ideal screening tool (Morris, 1991; Herman et al., 2013; Paleari et al., 2021). Mathematically, the Morris method employs a ‘one-at-a-time’ (OAT) sampling design across an N-dimensional unit hypercube. Each of the N parameters is sampled from a set of p discrete levels: $\{0,\frac{1}{p-1},\frac{2}{p-1},\ldots ,1\}$ , establishing a step size of $\Delta =\frac{1}{\left(p-1\right)}$ (Paleari et al., 2021). The model is evaluated along trajectories with N+1 points. For each point, a single parameter is shifted by Δ and the resulting ‘elementary effect’ ( $EE_{i}$ ) for the i -th parameter (Raj et al., 2024) is calculated as

$$EE_{i}=\frac{Y\left(x_{1},\ldots ,x_{i}+\Delta ,\ldots ,x_{N}\right)-Y\left(X\right)}{\Delta }$$ (1)

where $X=\left(x_{1},\ldots x_{N}\right)$ is the vector of model parameters and $Y\left(X\right)$ is the corresponding model output vector (Paleari et al., 2021; Raj et al., 2024). Since a single trajectory provides only a local measure dependent on the starting point, we evaluated 100 different trajectories ( r=100 ) to obtain a robust global estimate. With 20 different parameters ( N=20 ) across various categories (see Table 2), the prescreening required a total of $r\cdot \left(N+1\right)=2100$ model runs ( p=4 ). Both model endpoints, PEC_max_ and LP_50_, are compared based on their sensitivity results and interaction potential by using the mean (µ), and standard deviation (σ) of the elementary effects (Equation 1) (Tsvetkova and Ouarda, 2021). The mean µ indicates the overall first-order importance, whereas the standard deviation σ reflects the presence of non-linear effects and interactions (Paleari et al., 2021). We use µ* (the mean of absolute values) for ranking to avoid cancellation by negative effects (Raj et al., 2024). Parameters with low µ* and σ values were discarded for further analyses. This pre-screening uses the medium-kinetics parameter set for the GUTS-RED-SD model to rank parameters by their sensitivity to model output (Sarrazin et al., 2016). However, the results were checked for both fast and slow kinetics. It should be considered here that sensitivity is determined by the relative change in output in response to variations in input values, as well as the potential for interactions between these parameters. Therefore, results depend heavily on the selected parameter ranges.

### 2.4 Sobol sensitivity analysis

To refine the GSA, the Sobol method is applied to four parameters previously identified as sensitive during the Morris analysis. The variance-based Sobol sensitivity analysis employs a probabilistic framework to decompose the total variance of the model output into fractions attributable to individual inputs and their interactions. It varies all parameters simultaneously across their entire specified ranges. To implement this, the input space is mapped to a unit hypercube and sampled using the Saltelli approach (Saltelli, 2002; Saltelli et al., 2010), a Monte Carlo-based method that generates a structured set of points to efficiently explore the parameter space (Sobol, 2001). It generates two base sample matrices and derived hybrid matrices, from whose model outputs the Sobol’ indices are estimated. We assume a uniform distribution of values within the defined parameter ranges. Whereas the first-order Sobol index $S_{1}$ represents the main effect of a parameter $x_{i}$ ​ on the output variance $Var\left(Y\right)$ , this index fails to capture the interaction effects resulting from the simultaneous variation of multiple parameters. To address this, the total-order sensitivity index $S_{T_{i}}$ , is employed to quantify the total variance contribution of $x_{i}$ ​, encompassing both its primary effect and its interactions with all other input variables. It is defined as the ratio of the expected value of the conditional variance to the total variance:

$$S_{T_{i}}=\frac{{E_{X}}_{∼i}\left[Var_{X_{i}}\left(\left.Y\right|X_{∼i}\right)\right]}{Var\left(Y\right)}$$ (2)

where $X_{∼i}$ denotes all parameters except $x_{i}$ (Puy et al., 2022). To visualize the ranking of sensitivity, we calculated the relative proportion of the total-order Sobol index of one variable *i* (Equation 2) to the sum of all four Sobol indices for the selected parameters *J* (Equation 3). In doing so, we standardize the Sobol indices and can draw conclusions about the relative importance of the parameters; however, we deviate from the original definition of the Sobol indices as a proportion of the variance.

$$S_{T_{i,rel}}=\frac{S_{T_{i}}}{\sum_{j}S_{T_{j}}},j\in J$$ (3)

With varying four parameters, we quantified the Sobol indices with a base sample size of 128. Including second-order sampling, this results in 1280 model runs per analysis (for some analyses we extended to 256 samples to improve results by narrowing confidence intervals). Hereby, we perform Sobol analyses with varying Koc values to investigate the influence of sorption behavior on parameter sensitivity. Koc is varied logarithmically between 10 and 10000 in 30 steps. For each Sobol analysis, Koc values are varied over the range of 0.5 to 2 times the nominal value. As we were focusing on sensitivity ranking without assigning too much importance to the exact index values, this small sample size allows for a time-efficient analysis. For a more precise analysis of the Sobol indices, we recommend increasing the sample size.

## 3 Results

### 3.1 Morris screening results

The Morris analysis identified water depth as the most sensitive parameter in terms of the risk characterization based on LP_50_ values. While catchment area and Koc exhibited higher standard deviations and therefore suggested a greater potential for non-linear interactions, their mean absolute elementary effects (µ*) were lower than those of water depth. These results indicate that the risk is primarily driven by the dilution capacity associated with water depth, the pesticide loading from the surrounding catchment, and the chemical affinity of the pesticide for the soil. Water solubility and degradation rates also demonstrated high sensitivities (Figure 1). Conversely, sediment parameters and certain water body descriptors, such as length and width of the pond, exhibited negligible influence on LP_50_ values (SI, Supplementary Table S2). Based on these findings, we selected water depth, catchment size, Koc and DT_50_ in water for subsequent Sobol analyses. DT_50_ in soil was omitted as it is only included in PRZM. Water solubility was excluded because DT_50_ determines temporal resolution more than static water solubility, which reaches equilibrium quickly. Although the application date was identified as an influencing factor, it was excluded due to constraints in the R1 pond scenarios for the selection of winter cereals. The influence of application date is strongly coupled with meteorological data, which we did not vary in our analyses.

**FIGURE 1:**
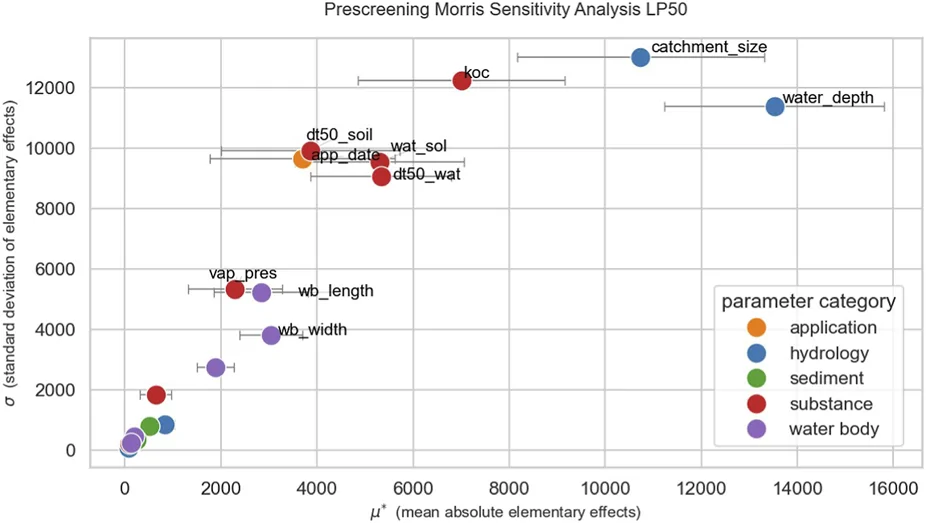
Scatter plot of sensitivity indices from Morris analysis for LP_50_ values.

Compared to the parameter influence on PEC_max_, water depth, catchment size and Koc remained the most sensitive parameters (see SI, Supplementary Figure S1). Additionally, water body width was identified as a sensitive parameter for peak concentrations. This suggests that the PEC_max_ is primarily determined by dilution and the magnitude of pesticide loading from the catchment. Since peaks typically occur shortly after application, degradation rates are not important. Notably, Koc exhibited the highest σ relative to µ* for PEC_max_, indicating that its influence is highly dependent on other system parameters.

In summary, Koc is a key factor influencing both LP_50_ and PEC_max_, highlighting the important role of sorption processes in aquatic systems. However, a clear discrepancy exists regarding degradation parameters: while DT_50_ is significant for long-term risk assessment (LP_50_), it is negligible for PEC_max_ (SI, Supplementary Table S1). Furthermore, the sensitivity shifts across environmental descriptors. LP_50_ is more influenced by the pesticide loading from catchments, whereas PEC_max_ is strongly influenced by water depth and water body width.

### 3.2 Sobol indices with varying Koc

The total-order Sobol indices (S_T_) reveal that water depth and catchment size most strongly influence the variance of PEC_max_ across different Koc value ranges. The sensitivity of water depth is highest at low Koc​ values and remains a dominant driver of uncertainty across the entire Koc range, although its influence decreases strongly for Koc values up to 20. Catchment size shows a moderate and relatively stable influence, whereas its influence is very low for small Koc values (Figure 2). In contrast, the influence of Koc is most pronounced at low to mid-range values (Koc<100) and decreases significantly as Koc​ increases (100<Koc<1000). For higher Koc values, S_T_ increases again, indicating that higher sorption potential influences concentration peaks (Figure 2). The DT_50_​ in water has a negligible impact on PEC_max_​ across all tested Koc​ values (for more information on figure interpretation, see SI, Supplementary Figure S2).

**FIGURE 2:**
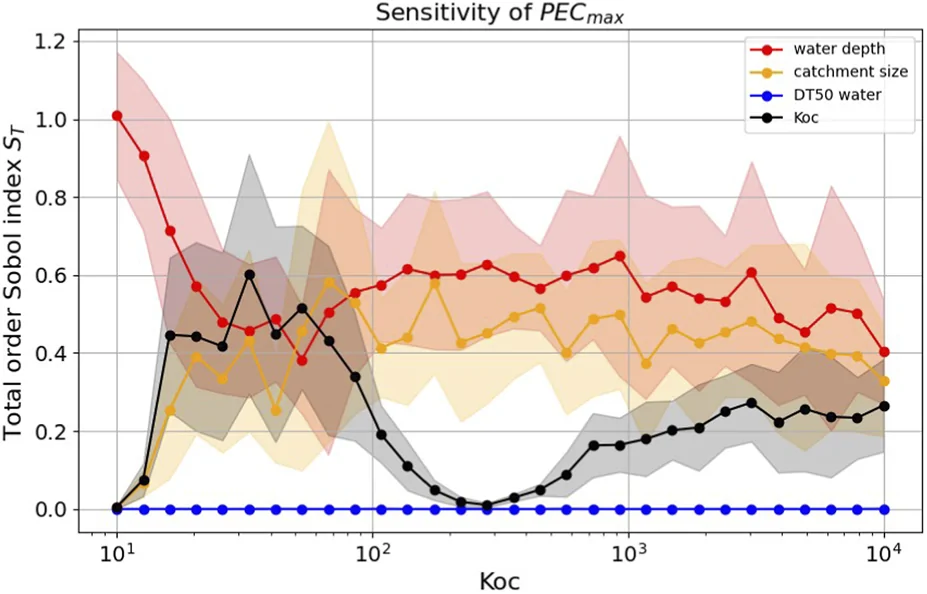
Sensitivity analysis of PEC_max_ with varying Koc value ranges. The points represent the calculated total-order Sobol indices (S_T_), which have been connected for visualization purposes. Shaded areas represent the 95% confidence intervals for each index.

In contrast, catchment size is the dominant driver of LP_50_ variance for medium and high Koc values. While water depth mainly influences the predicted risk for low Koc values, Koc values themselves showed similar influence on LP_50_ as on PEC_max_. Total sensitivity indices for DT_50_ remain low across the investigated Koc range, but show slightly elevated values and larger fluctuations than for PEC_max_ (Figure 3). This leads to the conclusion that the loading from surrounding catchments is the most important parameter for the risk prediction. However, water depth is important if Koc values are low and the influence of degradation times on LP_50_ is limited, suggesting that risk is mainly driven by transport processes and peak concentrations in water. Nevertheless, we identified degradation times as relevant in the previous Morris screening. Sobol results revealed only that water depth and catchment size are more important in comparison to DT_50_ times, which should not lead to omitting degradation in future exposure modeling.

**FIGURE 3:**
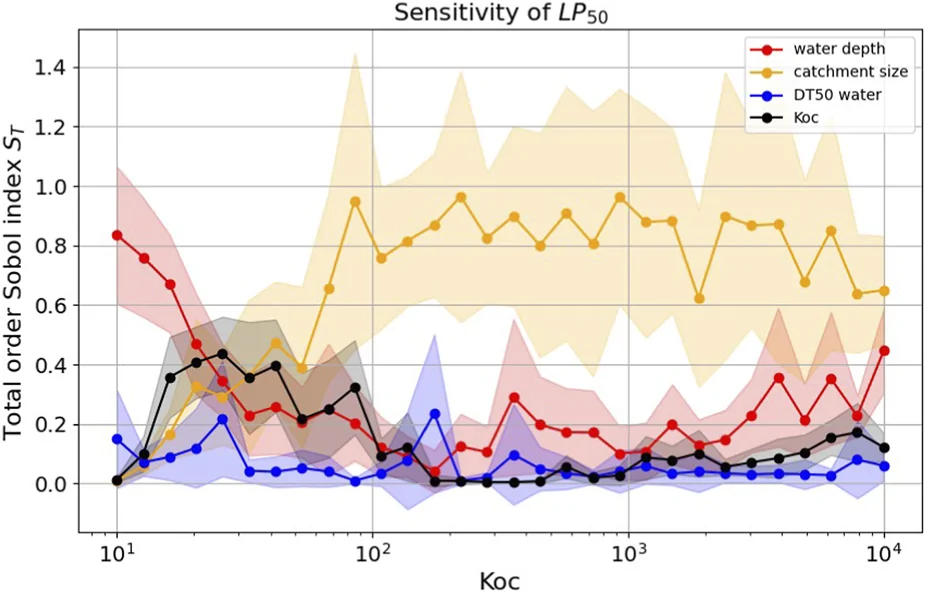
Sensitivity analysis of LP_50_ with varying Koc value ranges. The points represent the calculated total-order Sobol indices (S_T_), which have been connected for visualization purposes. Shaded areas represent the 95% confidence intervals for each index.

### 3.3 Influence of kinetics in GUTS-RED-SD

We applied the same GSA for three different kinetics, represented by varying *kd* and *m* parameters. As this affects only the GUTS model, the change in kinetics influences LP_50_ values but not PEC_max_ (Figure 2; SI, Supplementary Figures S3, S4). The general sensitivity ranking based on relative Sobol indices (Equation 3) remains the same for all kinetics, but we can show that degradation becomes more important for slower kinetics (Figure 4). Water depth is the most influential parameter on LP_50_ variance for lower Koc values, whereas the catchment size influences the risk predictions more for pesticides with higher sorption behavior. All three kinetics showed an increased influence of Koc values between 10 and 100 and nearly no influence between 100 and 1000. The influence of degradation time depends on the kinetics. For fast kinetics, risk is mainly driven by the peak concentrations, and high concentrations lead to fast damage dynamics (SI, Supplementary Figures S4, S6), whereas slow kinetics are more influenced by degradation rates (SI, Supplementary Figures S3, S5).

**FIGURE 4:**
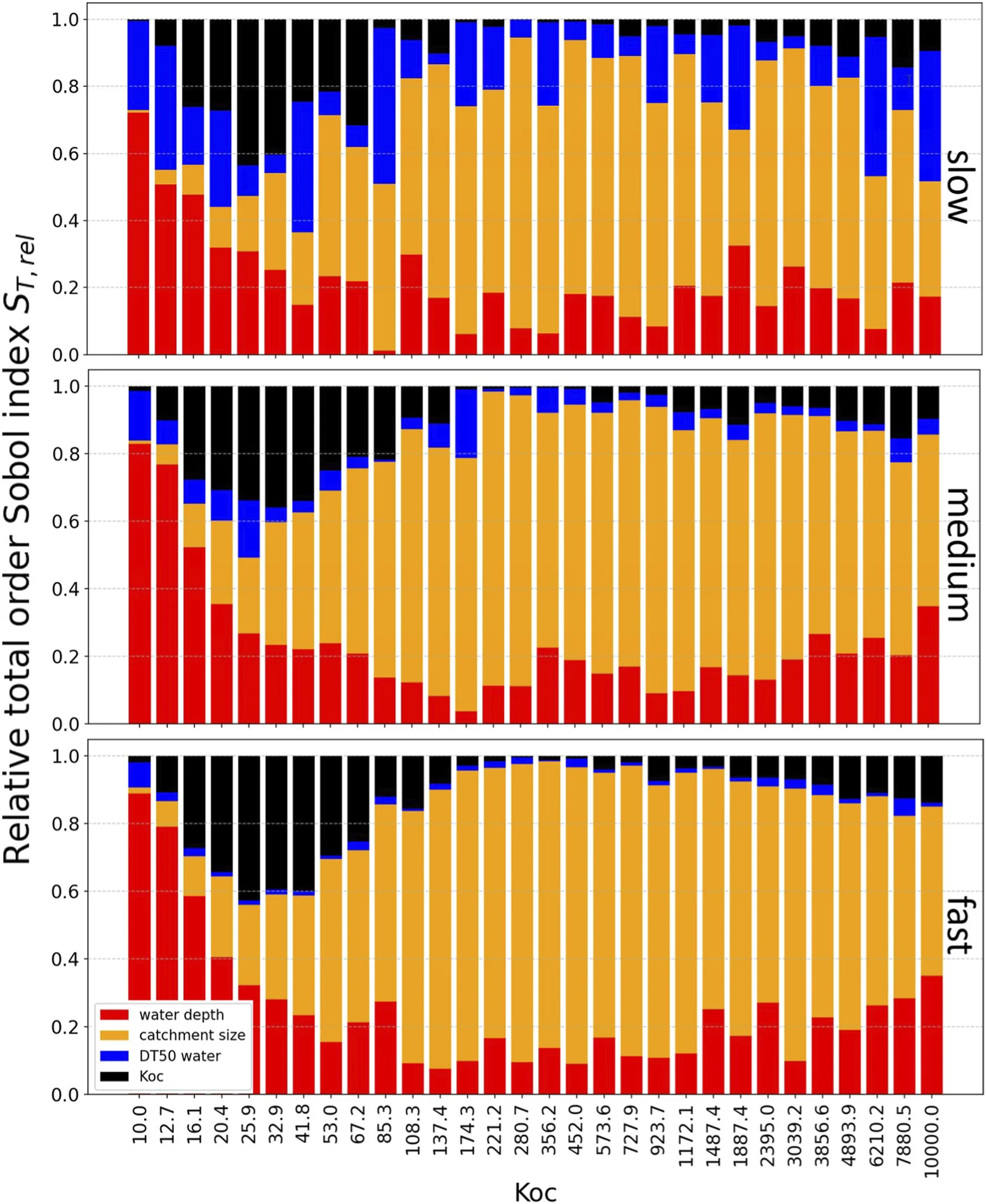
Relative sensitivity of the four selected parameters water depth, catchment size, DT_50_ in water and Koc for the three TKTD kinetics.

### 3.4 Results interpretation

Substance-specific parameters such as Koc and DT_50_ that can be determined via laboratory studies for regulatory purposes are well known, while environmental parameters, such as hydrological or soil characteristics, show site-specific variability. The preliminary Morris analysis indicates that sediment parameters are less important for model-based ERA, whereas the size of the water body and the catchment area strongly influence risk predictions for aquatic organisms.

For lower Koc values, water depth is more important than for higher Koc values, where pesticides tend to bind more strongly to the soil or sediment, thereby reducing their mobility in water. Furthermore, we observed that degradation (DT_50_) becomes more important for slower kinetics within the organisms: if a pesticide is more persistent in water and an organism exhibits slow kinetics, the organism may experience prolonged exposure and damage accrual. In general, dilution (e.g., via increased water volume due to greater water depth), degradation in water, and rapid runoff during rainfall events are more important for substances with low Koc values, reflecting their higher mobility and lower sorption. With fast kinetics, degradation has little influence on LP_50_. In such cases, dilution is primarily relevant during the initial exposure phase, and persistence of a pesticide is less important than high peak concentrations because of the rapid TKTD processes within the organism.

The divergent sensitivity profiles of peak concentrations versus LP_50_​ demonstrate that although peak concentrations are a major driver of risk, they do not exclusively determine the ecological risk on non-target organisms. The influence of Koc is the same for PEC_max_ and LP_50_, whereas the other parameters also influence the temporal dynamics of water concentrations, which are not captured by the maximum peak concentration alone but by the time-resolved LP_50_ values.

## 4 Discussion

In this study, we identified sensitive parameters by linking an exposure model to an effect model. However, our results are heavily dependent on the assumptions made. The choice of parameter ranges affects sensitivity by relating the variance of the model results to that of the varied input parameters. Our model assumptions and fixed parameters certainly also play a role and should be considered when interpreting the results. Furthermore, the variability assumed in our sensitivity analyses does not always reflect the natural variations observed in the real world. However, even if these results are more indicative and should be refined in subsequent analyses, this integrated approach provides a framework for joint exposure-effect assessments and suggests potential model simplifications.

### 4.1 Model assumptions and limitations

Our analysis focused on the R1 FOCUS pond scenario for Central Europe and is based on a treatment of winter cereals with an application dose of 1000 g/ha. While we attempted to cover different pond scenarios by varying hydrological parameters, such as pond size or proportion of organic matter, climatic and topographic parameters remained constant. It is acknowledged that stream and ditch scenarios would offer additional insights because pesticide transport and flow rates become more important here than in ponds (moving in water body; FOCUS, 2001). Furthermore, we considered only runoff and spray-drift as primary transport processes from field to water by the PRZM model for the R1 pond scenario. Drainage, which is covered by the MACRO model (Jarvis, 1994) and different FOCUS scenarios, was not considered and could be an important transport pathway for pesticide fate (FOCUS, 2001). Koc may have a greater influence on drainage and leaching than on runoff because, while pesticides with a high Koc can transfer to surface water via erosion (e.g. particle-bound transport), they cannot be transported via drainage. While TOXSWA allows for application series with individually determinable intervals, this study assumes only single applications to maintain a clear focus on the transport processes within FOCUS Step 3. Incorporating multiple applications would introduce complex accumulation dynamics, which could make comparisons more difficult. More applications usually lead to higher PECs and thus to greater risks. A similar trend is expected for the application dose: We assume a linear relationship here (higher application doses lead to higher risks to organisms), so we excluded this parameter from sensitivity analysis and focused on parameters with more interaction potential. In contrast, the application date remains a critical variable, as it is intrinsically linked to the crop growth stage and time-varying weather data. Morris analysis results revealed that the application date is among the top ten sensitive parameters (Figure 1; SI, Supplementary Table S2). However, we believe that farmers check the weather forecast before applying pesticides to avoid unnecessary runoff from rainfall, which could reduce effectiveness and pose economic risks.

For the model-based effect assessment, the study uses generic GUTS kinetics. Although three different kinetics were considered (medium, slow, fast), the calibration parameter sets are not directly linked to species. Using species-specific parameterizations (e.g. for *Daphnia magna*, *Gammarus pulex*, standard fish species) would enhance transferability to regulatory contexts.

An important factor in sensitivity analysis is the number of 1280 model runs (in some analyses exceeded to 2560 runs) for each GSA. Based on the Sobol indices and their 95% confidence intervals, we see strong uncertainties in the sensitivity calculation due to overlapping confidence intervals. For future GSAs, we recommend increasing the number of model runs to reduce these uncertainties in sensitivity calculations and to define convergence criteria in advance (Sarrazin et al., 2016). Ideally, calculation time could be reduced by using high-performance computers.

Despite these constraints, the methodology provides a framework for streamlining future risk assessments by identifying and prioritizing the most sensitive parameters. Analyses can be extended to other scenarios, treatment schemes and transport pathways.

### 4.2 Environmental variability

Ecological systems are characterized by high variability, which frequently translates into significant uncertainty in model input parameters (Regan et al., 2003). Due to the immense dimensionality of ecological systems and the limited availability of high-resolution data, models cannot realistically capture every environmental variable (Gerhard et al., 2023). To balance computational feasibility and regulatory practicality, models often rely on simplified representations of the environment and representative scenarios to capture the most critical conditions. For instance, FOCUS employs ten scenarios derived from representative European datasets to predict worst-case risks (FOCUS, 2001). With the environmental conditions across the different scenarios, the 98th percentile of agricultural runoff land and the 99th percentile of drained agricultural land in the EU are captured (FOCUS, 2001). In total, 32.9% of all agricultural land in the EU is covered by the ten scenarios (FOCUS, 2001). All FOCUS scenarios incorporate agro-environmental characteristics, such as weather data (temperature, precipitation) and geographical specifics (e.g., slope, soil drainage, land cover, and cropping), as well as detailed data on soil, sediment, and water body definitions (FOCUS, 2001). Although these parameters are critical for simulating transport processes and predicting the environmental fate of pesticides, they are characterized by both spatial heterogeneity across locations and temporal heterogeneity. The latter is primarily influenced by seasonality, weather, and anthropogenic activities, such as pesticide treatments at different crop growth stages. Consequently, these dynamics alter both the description of the modeled environmental compartment and the associated hydrological regimes (Marum et al., 2026). This may result in scenarios that omit rare but impactful environmental combinations or extreme events (e.g. heavy rainfalls following a long dry spell shortly after pesticide application) and therefore can lead to underpredictions of risk (Knäbel et al., 2012, Knäbel et al., 2014; Scharlach et al., 2026).

#### 4.2.1 Sensitive parameters are potential risk drivers

In this context, sensitivity analyses are a useful tool for identifying environmental parameters that have the most significant influence on the model’s output. This allows researchers to determine whether the chosen scenarios are truly representative of the risk and to quantify the uncertainty associated with the model’s predictions. It ensures that the risk assessment is robust and that the most critical parameter ranges are considered in the scenarios. From our Morris prescreening, we learned that some parameters describing the water system are very sensitive to parameter changes (e.g. water depth, catchment size), whereas others could be changed without influencing risk predictions much (e.g. water body length and width). We also identified important substance properties, such as Koc, degradation rates and water solubility, which are strongly linked to transport mechanisms. The distribution and persistence of pesticides in water are crucial to their uptake by the organism and the resulting damage. Others, such as molar mass or vapor pressure, which are also required in the FOCUS Step 3 model, are substance properties that do not really affect model results. If substance properties are not directly linked to transport and degradation mechanisms, it can be assumed that they have only minor influence on the fate and risk predictions. When considering degradation, it is important to remember that metabolites may also affect organisms. As the focus here is solely on generic parent substances, degradation products are not considered, which means that potential risk could be higher. Also, sediment parameters are identified as insensitive, indicating a future simplification by neglecting precise sediment characterizations for the risk to aquatic organisms. However, the sediment also provides habitat for some organisms. For benthic organisms, knowledge of sediment contamination is important for the subsequent risk assessment (EFSA, 2015). Unlike previous studies, we could not prove that chemical properties contribute more to variance in PEC than variability in transport factors and landscape characteristics (Hertwich et al., 1999). For the Morris and Sobol analyses, we identified the catchment size as one of the main drivers for elevated PECs and risk quantification.

#### 4.2.2 Parameter spaces influence sensitivities

A critical aspect of GSA is the definition of the input parameter space. Since sensitivity indices are properties of the model within a specified range rather than intrinsic model properties, the choice of parameter bounds directly influences the resulting sensitivity rankings.

We derived parameter ranges from the FOCUS scenarios, which cover a wide range of potential environmental conditions and are based on representative European datasets and monitoring sites, supplemented with data from literature (see Chapter 2.2). The choice of parameter bounds is a balancing act: ranges that are too narrow may hide interactions, while those that are too wide may overstate non-linearities and diverge from realistic environmental conditions. This dependence is evident in both employed methods: in the Morris analysis, the step size Δ is proportional to the defined range (see Chapter 2.3), and in Sobol analysis, the decomposition of output variance is a function of the input variable distributions (see Chapter 2.4). Consequently, the resulting indices for both methods are intrinsically linked to the chosen parameter spaces.

The high sensitivity of the catchment size may therefore also be due to the chosen parameter bounds, which are more variable in our study compared with the standard assumption of 0.45 ha (see Table 2). Different sensitivities can be calculated using different parameter selections or parameter ranges. For this reason, any generalization of the present findings must be made with caution, and we recommend a sensitivity analysis on parameter definition in future investigations.

#### 4.2.3 Risk drivers in ERA

Even though the models identify clear parameters that can be regarded as risk drivers, a critical distinction must be made between model sensitivity and real-world variability. There is often a trade-off between the range of variability that a model can computationally cover and the actual heterogeneity observed in nature (Gerhard et al., 2023). It is important to note that a parameter identified as sensitive through sensitivity analysis is not necessarily the most variable parameter in the field. A model may be highly sensitive to a specific input, meaning small fluctuations in that value lead to significant changes in the predicted risk, even if that parameter remains relatively stable in the real-world environment. This discrepancy suggests that high sensitivity does not always equate to high ecological uncertainty, but rather reflects the model’s internal responsiveness to that specific variable. Conversely, parameters that exhibit high natural variability may have a negligible impact on the model output, rendering their precise measurement less critical for risk prediction. As highly variable parameters are often difficult to measure, such a finding would reduce uncertainties in model predictions. Therefore, parameters identified as sensitive must always be considered in relation to reality. This distinction also reveals a potential limitation in regulatory frameworks: if model ranges are chosen for conservatism rather than biological realism, the sensitivity analysis may prioritize ‘worst-case’ parameters over the most frequent parameter ranges in the field. A better understanding of natural variability can only be achieved through increased monitoring (La Cecilia et al., 2021; Scharlach et al., 2026). Integrating empirical data enables the transition from broad, uniform parameter ranges to more accurate probability distributions that more realistically reflect reality. In this analysis, it was assumed that the values were uniformly distributed across the respective parameter ranges. This means that every value within the defined ranges is equally likely. To better reflect reality, one could assume a normal distribution and thus give greater weight to more probable values in the analysis. In addition, sensitivity analyses can be conducted using data from pesticide databases and monitoring data to ensure that they are grounded in ecological reality rather than computational convenience.

Identifying the four sensitive parameters (water depth, catchment size, Koc and degradation time) could inform concrete regulatory priorities, but the implications differ by parameter type. Koc and DT_50_ are substance properties already determined experimentally for an ERA dossier. Our results support keeping the effort there, while insensitive substance and sediment inputs could be set to conservative default values. Water depth and catchment size are scenario properties that vary widely in the field and may hence not effectively differ between the pesticides being assessed. High sensitivity to these parameters argues for defining them carefully and conservatively at the scenario and guidance level, informed by landscape and monitoring data, rather than opening them to case-by-case refinement in dossiers, which risks adding workload and delaying decisions (Duquesne et al., 2026). For monitoring, our results suggest that water body dimensions and the size and land use of the contributing catchment are valuable metadata when measured concentrations are used to evaluate exposure models. As we analyzed a pond scenario, this remains to be confirmed for streams. To support any of these uses, the results must be linked more closely to reality, through adjusted parameter ranges (see Section 4.2.2) or species-specific parameterization.

### 4.3 Implications for risk assessment

The primary objective of ERA is to identify potential risks to protect human and animal health, as well as the environment (Regulation (EC) No. 1107/2009, Art. 1). To ensure that ERA remains manageable for regulators, model complexity must be carefully balanced, which is one of the reasons ERA follows a tiered approach starting with simple assumptions and increasing complexity if needed. A thorough understanding of uncertainty is therefore crucial for evaluating the robustness of model-based decisions and strategically allocating resources (Hertwich et al., 1999). Sensitivity analysis facilitates this by identifying parameters with minimal impact on model predictions. This identification can replace costly and time-consuming laboratory and monitoring data with default values, or even eliminate redundant processes to simplify the models. For instance, our results indicate that sediment parameters are not sensitive for risk predictions on aquatic organisms. Detailed information on sediment characteristics, e.g., organic matter mass content or bulk density, is not useful for effective risk prediction, as these parameters only marginally influence risk predictions. Conversely, the identification of sensitive parameters allows targeted monitoring and optimization of worst-case scenarios, ensuring that conservative parameter values are applied where they matter most. We identified dilution, loading from the catchment and sorption behavior as important processes for risk prediction. Consequently, we conclude that a precise description of the water body size (especially water depth), the pesticide treatment on adjacent fields and the Koc are important parameters that can be verified by monitoring. However, sensitivity analysis so far has only revealed the influence of a parameter change on the model outcome, but not concrete parameter ranges that elevate the risk. Therefore, subsequent analyses are required to define worst-case parameter ranges.

Our results suggest that model complexity can be reduced by focusing on a few highly sensitive parameters while maintaining insensitive parameters at realistic, generalized default values. Parameters whose modification has little impact on the model results should require minimal effort to incorporate into the model, in order to maintain an appropriate cost-benefit ratio in the ERA. Finally, it should be noted that a comprehensive risk assessment involves not only specific scenarios and substance information but also regulatory aspects (e.g., threshold values). Standard lower-tier ERA compares maximum, or under certain conditions time-weighted average PECs with effect thresholds (Ashauer et al., 2006; Junghans et al., 2006), while time-variable exposure can be addressed in refinements, including TKTD modeling (EFSA, 2013; EFSA et al., 2018). Our analysis shows that a peak-based and a time-resolved metric can rank the same input parameters differently. A sensitivity analysis is, however, internal to the model and hence cannot alone show which metric better predicts effects in the field. This requires benchmarking TKTD-based predictions against monitoring data and simpler measures of toxic pressure, in line with the call to test the predictive power of models of differing complexity against field data (Axelman et al., 2024). A first benchmark for small streams found that TKTD-derived LP_50_ explained macroinvertebrate community composition in water streams no better than peak toxic pressure (Siddique et al., 2026). It remains to be tested whether this also holds for ponds and daily-resolved exposure, as in FOCUS Step 3. Pulsed exposure may involve cumulative or delayed effects that would be missed by considering only PEC_max_ (Ashauer and Escher, 2010). Nevertheless, our results provide insights into the interaction effects arising from different parameter combinations.

## 5 Conclusion

Our results demonstrate that Koc, water depth, and catchment size mainly influence risk predictions. Consequently, we conclude that a precise description of the water body size (especially water depth), combined with knowledge about the sorption behavior and application dose in adjacent fields, is more important than a detailed characterization of sediments, buffer zones, or soil properties.

A comparison of parameter influences on PEC_max_ and LP_50_ revealed that water body characteristics are more important for predicting peak concentrations (PEC_max_), whereas degradation processes and pesticide persistence in water are more important for risk prediction (LP_50_). In contrast, soil and sediment properties were found to be relatively insensitive for both model outcomes. Although application doses and timing are recognized as key factors in ERA, they were not included in our analysis due to their linear influence on PECs, as well as the influence of weather conditions, which farmers usually take into account when planning applications.

In general, parameters that influence PEC_max_ also influence LP_50_ values, suggesting that risk is affected by high peak concentrations. However, the influence of degradation processes and the kinetic speed in the organisms on risk predictions can also be observed. For fast kinetics, degradation rates play a smaller role than for slower kinetics, where the residence time of pesticides in the water is more important. When analyzing the temporal resolution of concentrations in water bodies, consideration is given not only to the maximum concentration but also to the duration of concentration peaks and the temporal dynamics of damage processes within organisms. Both are reflected in the GUTS model, which thus goes one step further than the current ratio-based effect prediction in ERA.

Identifying the most influential parameters for risk predictions and time-resolved concentration patterns could lead to simplified models by reducing the number of input parameters and setting less sensitive values to conservative default values.

## Data Availability

The original contributions presented in the study are included in the article and Supplementary Material, further inquiries can be directed to the corresponding author.
