# Supplementary material for: What parameters really matter? Linking exposure and effect models to identify sensitive parameters for model-based aquatic risk predictions of pesticides
Source: Front Toxicol. 2026 Oct 27;8:1984119. doi: 10.3389/ftox.2026.1984119 (PMC13747304; doi:10.3389/ftox.2026.1984119)
Supplement: Supplementary file 1 [file DataSheet1.docx]

**Supporting Information**

12 pages

7 Tables

6 Figures

Contents

[A)	GUTS model framework	2](#_Toc242426769)

[B)	Morris – Prescreening	3](#_Toc242426770)

[C)	Sobol analysis	6](#_Toc242426771)

[Total-order sobol indices for PEC_max_ and LP_50_	7](#_Toc242426772)

[Visual representation of total-order sobol indices for PEC_max_	11](#_Toc242426773)

[Visual representation of total-order sobol indices for LP_50_	12](#_Toc242426774)

# A) GUTS model framework

The General Unified Threshold model of Survival (GUTS) belongs to the group of TKTD models that simulate how time-resolved exposure patterns lead to toxic effects in organisms. These models integrate toxicokinetics (TK), which cover bioaccumulation, distribution, biotransformation, and elimination of chemicals, with toxicodynamics (TD), which describes subsequent damage accrual and recovery. When data on body residues are unavailable, the TK and TD components cannot be modelled independently, so GUTS-reduced (GUTS-RED) models are employed instead, effectively integrating TK and TD into a simplified framework. We used GUTS-RED to include PECs from FOCUS Step 3 as external concentrations.

GUTS distinguishes between two death mechanisms: stochastic death (SD) and individual tolerance (IT). We only included SD in our analysis, in which all individuals are assumed to be identical, and the probability of death increases continuously with chemical stress.

The mathematical description of GUTS-RED-SD can be simplified to:

$$\frac{dD_{w}}{dt}=k_{d}(c_{w}-D_{w})$$

$$h_{z}=b\cdot max(0,D_{w}-m)$$

$$\frac{dS}{dt}= -h_{z}\cdot S$$

where $k_{d}$ is the dominant rate constant, $c_{w}$is the external concentration (generated by FOCUS Step 3), $b$ represents the killing rate and $m$ describes the median effect threshold. With these parameters, damage ($D_{w}$), hazard rate ($h_{z})$ and survival probability ($S$) can be calculated.

To include different kinetics, we applied three different parametrizations by changing $k_{d}$ and $m$. The medium kinetic was taken from a generic substance reported in EFSA Sc. Opinion on TKTD models (Figure 8)^[1]^. Increasing the dominant rate constant $k_{d}$and at the same time decreasing the median threshold $m$, accelerates kinetic processes. Conversely, a decrease in $k_{d}$ accompanied by an increase in $m$slows them down.

The parameterized models were then used to calculate lethal profiles (LP_50_) for each time-resolved exposure pattern from FOCUS Step 3. LP_50_ values can be interpreted as multiplication factors that, when applied to an exposure profile, will result in 50% mortality within the population.

We performed all GUTS calculations with the open-source Python package ‘*memPyGUTS’* (version 1.7.0b2), publicly available on GitLab^[2]^. LP_50_ values were determined recursively using the LP*xEstimator* function in *memPyGUTS*.

# B) Morris – Prescreening

The Morris analysis is a computationally efficient global sensitivity analysis method that was used to identify the most sensitive parameters within the predefined parameter spaces.

N = 20 (number of parameters), r = 100 (number of trajectories), p = 4 (step size)

in total: 2100 model runs

µ: mean elementary effect

µ*: absolute of the mean elementary effect

σ: standard deviation of elementary effect

µ* confidence interval: the bootstrapped confidence interval (95%)

Table S1: Results for the Morris Sensitivity analysis on PEC_max_.

| **Parameter** | **µ** | **µ*** | **σ** | **µ***  **confidence interval** |
| --- | --- | --- | --- | --- |
| water_depth | -3.79 | 3.79 | 7.64 | 1.56 |
| Koc | -2.21 | 3.07 | 9.79 | 1.92 |
| catchment_size | 2.99 | 2.99 | 6.44 | 1.30 |
| wb_width | -2.10 | 2.10 | 6.24 | 1.16 |
| wb_length | -1.38 | 1.38 | 3.28 | 0.66 |
| app_date | -1.12 | 1.28 | 7.21 | 1.25 |
| dt50_soil | 0.76 | 0.76 | 1.75 | 0.34 |
| mass_ratio_oc | -0.22 | 0.22 | 0.50 | 0.09 |
| susp_solid | -0.18 | 0.18 | 0.66 | 0.11 |
| side_slope | -0.10 | 0.14 | 0.29 | 0.05 |
| water_width | 0.08 | 0.08 | 0.21 | 0.04 |
| vap_pres | -0.08 | 0.08 | 0.41 | 0.08 |
| wat_sol | 0.07 | 0.07 | 0.27 | 0.05 |
| inflow_pond | -0.07 | 0.07 | 0.17 | 0.03 |
| dt50_wat | 0.07 | 0.07 | 0.13 | 0.03 |
| om | -0.03 | 0.03 | 0.06 | 0.01 |
| theta_sat | -0.02 | 0.02 | 0.03 | 5E-3 |
| dt50_sed | 0.01 | 0.01 | 0.02 | 4E-3 |
| bd | -0.01 | 0.01 | 0.03 | 4E-3 |
| mol_mas | -5E-3 | 5E-3 | 0.02 | 5E-3 |

Figure S1: Visualization of Morris sensitivity results on PEC_max_.

Table S2: Results for the Morris sensitivity analysis on LP_50_ with medium kinetics.

| **Parameter** | **µ** | **µ*** | **σ** | **µ***  **confidence interval** |
| --- | --- | --- | --- | --- |
| water_depth | 13527.62 | 13527.62 | 11383.93 | 2289.76 |
| catchment_size | -10691.91 | 10741.31 | 13015.97 | 2570.80 |
| Koc | 1277.28 | 7008.13 | 12253.98 | 2153.48 |
| dt50_wat | -5330.97 | 5330.97 | 9ß72.09 | 1464.48 |
| wat_sol | -5302.60 | 5302.60 | 9558.08 | 1753.49 |
| dt50_soil | -3862.74 | 3862.74 | 9932.80 | 1852.84 |
| app_date | 3514.25 | 3691.39 | 9648.77 | 1930.72 |
| wb_width | 2915.21 | 3046.08 | 3811.14 | 655.17 |
| wb_length | 2274.01 | 2839.51 | 5214.84 | 981.26 |
| vap_pres | 2292.63 | 2292.63 | 5330.67 | 979.45 |
| side_slope | -1126.81 | 1885.65 | 2754.23 | 390.16 |
| inflow_pond | 836.55 | 836.55 | 861.79 | 192.41 |
| mol_mas | 642.94 | 642.94 | 1837.16 | 325.23 |
| om | 503.73 | 503.73 | 785.90 | 152.31 |
| theta_sat | 241.29 | 241.29 | 331.29 | 55.30 |
| bd | 161.79 | 161.79 | 287.87 | 51.22 |
| susp_solid | -186.45 | 186.45 | 436.87 | 86.94 |
| mass_ratio_oc | -119.44 | 119.44 | 224.57 | 41.19 |
| water_width | -69.69 | 69.69 | 77.21 | 15.20 |
| dt50_sed | -97.00 | 97.00 | 201.17 | 35.46 |

Additionally, Morris analysis was performed for slow and fast kinetics to demonstrate that the selected parameters for subsequent analyses are also valid across different kinetics.

Table S3: Results for the Morris sensitivity analysis on LP_50_ with slow kinetics.

| **Parameter** | **µ** | **µ*** | **σ** | **µ***  **confidence interval** |
| --- | --- | --- | --- | --- |
| water_depth | 82333.83 | 82333.83 | 73336.86 | 14260.85 |
| catchment_size | -64547.31 | 64971.16 | 83538.32 | 15690.09 |
| Koc | 15964.49 | 32894.15 | 53584.01 | 8968.38 |
| dt50_wat | -44050.62 | 44050.62 | 76306.35 | 13260.04 |
| wat_sol | -38991.05 | 38991.05 | 68447.11 | 12971.60 |
| dt50_soil | -28595.20 | 28595.20 | 43325.75 | 8665.59 |
| app_date | 12316.60 | 13939.54 | 39256.91 | 8032.74 |
| wb_width | 12854.18 | 14593.50 | 24296.15 | 4435.60 |
| wb_length | 15883.60 | 20098.14 | 33290.78 | 5530.89 |
| vap_pres | 21612.56 | 21612.56 | 52825.85 | 10058.69 |
| side_slope | -4533.05 | 8039.57 | 14388.40 | 2344.66 |
| inflow_pond | 5626.51 | 5626.51 | 6312.30 | 1374.68 |
| mol_mas | 5683.11 | 5683.11 | 15866.73 | 3188.87 |
| om | 4846.68 | 4846.68 | 7420.16 | 1506.43 |
| theta_sat | 2107.54 | 2107.54 | 2445.94 | 477.27 |
| bd | 1617.68 | 1671.68 | 2045.58 | 394.31 |
| susp_solid | -1196.01 | 1196.01 | 2675.37 | 488.96 |
| mass_ratio_oc | -1348.63 | 1348.63 | 2630.26 | 526.23 |
| water_width | -424.02 | 424.02 | 457.40 | 88.56 |
| dt50_sed | -868.04 | 868.04 | 1664.19 | 336.33 |

Table S4: Results for the Morris sensitivity analysis on LP_50_ with fast kinetics.

| **Parameter** | **µ** | **µ*** | **σ** | **µ***  **confidence interval** |
| --- | --- | --- | --- | --- |
| water_depth | 5847.96 | 5847.96 | 4218.09 | 871.40 |
| catchment_size | -3847.04 | 3855.65 | 4421.65 | 836.76 |
| Koc | 325.61 | 2347.85 | 4150.29 | 638.49 |
| dt50_wat | -1181.57 | 1181.57 | 1782.39 | 310.66 |
| wat_sol | -917.89 | 917.89 | 1792.86 | 334.79 |
| dt50_soil | -2360.64 | 2360.64 | 4005.52 | 769.52 |
| app_date | 1808.37 | 1990.32 | 3954.87 | 770.73 |
| wb_width | 989.51 | 1053.01 | 1482.73 | 302.87 |
| wb_length | 779.89 | 883.62 | 1396.58 | 249.22 |
| vap_pres | 489.23 | 489.23 | 1112.89 | 190.58 |
| side_slope | -511.09 | 734.19 | 1039.42 | 176.31 |
| inflow_pond | 230.77 | 230.77 | 314.30 | 66.42 |
| mol_mas | 114.26 | 114.26 | 315.90 | 62.32 |
| om | 228.92 | 228.92 | 435.46 | 85.62 |
| theta_sat | 46.93 | 46.93 | 63.98 | 12.33 |
| bd | 68.90 | 68.90 | 105.27 | 21.34 |
| susp_solid | -26.26 | 26.26 | 53.85 | 10.51 |
| mass_ratio_oc | -41.60 | 41.60 | 80.97 | 15.35 |
| water_width | -33.0 | 33.0 | 33.64 | 5.63 |
| dt50_sed | -43.15 | 43.15 | 144.87 | 28.11 |

# C) Sobol analysis

From the Morris prescreening, we identified the four most sensitive parameters and included them in a subsequent Sobol sensitivity analysis. Thereby, we varied Koc values from 10 to 1000 in 30 steps to investigate the influence of Koc on sensitivity ranking.

N = 4 (number of parameters), r = 128 or 256 (sample size)

in total: 1280 or 2560 model runs for each Sobol analysis

As Koc is also among the parameters of interest for the Sobol analysis, we varied its range from 0.5*Koc to 2*Koc.

Figure S2: Visualization of how to interpret the points of the Sobol analyses.

## Total-order sobol indices for PEC_max_ and LP_50_

Table S5: Total-order Sobol indices $S_{T_{i}}$ for slow kinetics. Grey values represent the 95% confidence intervals of the $S_{T_{i}}$. The values are rounded.

| **Koc** | **Model runs** | **PEC_max_** | | | | **LP_50_** | | | |
| --- | --- | --- | --- | --- | --- | --- | --- | --- | --- |
|  |  | **Water depth** | **DT50** | **Koc** | **area** | **Water depth** | **DT50** | **Koc** | **area** |
| **10.00** | 1280 | 1.02 (0.15) | 9E-5  (9E-5) | 0.005 (0.005) | 0.005 (0.004) | 0.59 (0.45) | 0.22 (0.11) | 0.005  (0.005) | 0.006 (0.007) |
| **12.69** | 1280 | 0.92 (0.21) | 2E-4  (2E-4) | 0.10  (0.09) | 0.08  (0.06) | 0.68 (0.25) | 0.49 (0.55) | 0.11 (0.06) | 0.06 (0.05) |
| **16.10** | 1280 | 0.70 (0.18) | 3E-4 (2E-4) | 0.35 (0.12) | 0.23 (0.18) | 0.43 (0.22) | 0.15 (0.17) | 0.23 (0.11) | 0.08 (0.06) |
| **20.43** | 1280 | 0.48 (0.35) | 1E-4 (8E-5) | 0.38 (0.24) | 0.25 (0.20) | 0.32 (0.20) | 0.29 (0.31) | 0.27 (0.25) | 0.12 (0.11) |
| **25.93** | 1280 | 0.44 (0.20) | 9E-5 (6E-5) | 0.58 (0.35) | 0.32 (0.19) | 0.39 (0.13) | 0.12 (0.10) | 0.56 (0.17) | 0.21 (0.08) |
| **32.90** | 1280 | 0.45 (0.45) | 8E-5 (6E-5) | 0.46 (0.23) | 0.42 (0.36) | 0.29 (0.15) | 0.06 (0.04) | 0.46 (0.17) | 0.33 (0.14) |
| **41.75** | 1280 | 0.38 (0.16) | 5E-5 (3E-5) | 0.50 (0.28) | 0.33 (0.21) | 0.17 (0.18) | 0.44 (0.43) | 0.28 (0.25) | 0.24 (0.21) |
| **52.98** | 1280 | 0.49 (0.29) | 2E-5 (2E-5) | 0.39 (0.17) | 0.39 (0.22) | 0.29 (0.25) | 0.09 (0.10) | 0.26 (0.17) | 0.58 (0.35) |
| **67.23** | 1280 | 0.48 (0.22) | 5E-5 (8E-5) | 0.39 (0.18) | 0.32 (0.14) | 0.30 (0.42) | 0.09 (0.13) | 0.44 (0.23) | 0.56 (0.27) |
| **85.32** | 1280 | 0.47  (0.16) | 2E-5 (2E-5) | 0.26 (0.13) | 0.48 (0.26) | 0.02 (0.14) | 0.66 (0.57) | 0.04 (0.23) | 0.71 (0.39) |
| **108.26** | 2560 | 0.52 (0.12) | 3E-5 (2E-5) | 0.16 (0.06) | 0.41 (0.13) | 0.45 (0.65) | 0.17 (0.23) | 0.10 (0.06) | 0.80 (0.28) |
| **137.38** | 1280 | 0.50 (0.19) | 4E-4 (8E-4) | 0.10 (0.07) | 0.48 (0.20) | 0.07 (0.17) | 0.01 (0.04) | 0.04 (0.11) | 0.28 (0.72) |
| **174.33** | 2560 | 0.61 (0.15) | 2E-4 (2E-4) | 0.06 (0.03) | 0.52 (0.15) | 0.07 (0.04) | 0.36 (0.38) | 0.01 (0.04) | 0.99 (0.51) |
| **221.22** | 1280 | 0.53 (0.16) | 2E-5 (2E-5) | 0.01 (0.01) | 0.49 (0.18) | 0.28 (0.40) | 0.28 (0.34) | 0.04 (0.04) | 0.92 (0.33) |
| **280.72** | 2560 | 0.58 (0.16) | 2E-4 (2E-4) | 0.01 (3E-3) | 0.49 (0.15) | 0.08 (0.04) | 0.05 (0.06) | 3E-3 (1E-3) | 0.88 (0.31) |
| **356.22** | 2560 | 0.58 (0.14) | 7E-5 (6E-5) | 0.03 (0.01) | 0.48 (0.12) | 0.07 (0.05) | 0.30 (0.45) | 0.01 (0.01) | 0.82 (0.38) |
| **452.04** | 2560 | 0.57 (0.14) | 7E-5 (9E-5) | 0.05 (0.02) | 0.50 (0.16) | 0.23 (0.13) | 0.07 (0.07) | 0.01 (0.01) | 0.99 (0.32) |
| **573.62** | 2560 | 0.57 (0.14) | 5E-5 (5E-5) | 0.08 (0.03) | 0.55 (0.14) | 0.21 (0.12) | 0.12 (0.20) | 0.02 (0.01) | 0.87 (0.30) |
| **727.90** | 2560 | 0.54 (0.13) | 6E-5 (6E-5) | 0.12 (0.04) | 0.48 (0.15) | 0.13 (0.07) | 0.07 (0.06) | 0.06 (0.02) | 0.89 (0.66) |
| **923.67** | 2560 | 0.55 (0.14) | 4E-5 (3E-5) | 0.16 (0.06) | 0.40 (0.13) | 0.09 (0.10) | 0.25 (0.17) | 0.02 (0.03) | 0.72 (0.63) |
| **1172.10** | 1280 | 0.50 (0.20) | 7E-5 (1E-4) | 0.19 (0.08) | 0.46 (0.21) | 0.30 (0.23) | 0.09 (0.08) | 0.07 (0.09) | 1.00 (0.58) |
| **1487.35** | 2560 | 0.57 (0.12) | 6E-5 (4E-5) | 0.20 (0.07) | 0.47 (0.13) | 0.27 (0.19) | 0.32 (0.27) | 0.08 (0.05) | 0.91 (0.33) |
| **1887.39** | 1280 | 0.51 (0.22) | 4E-5 (3E-5) | 0.19 (0.11) | 0.41 (0.18) | 0.60 (0.64) | 0.58 (0.79) | 0.04 (0.04) | 0.64 (0.23) |
| **2395.03** | 1280 | 0.61 (0.20) | 2E-4 (3E-4) | 0.27 (0.16) | 0.49 (0.17) | 0.11 (0.09) | 0.04 (0.04) | 0.05 (0.04) | 0.58 (0.40) |
| **3039.20** | 1280 | 0.52 (0.23) | 6E-4 (1E-3) | 0.34 (0.14) | 0.46 (0.21) | 0.33 (0.31) | 0.05 (0.03) | 0.07 (0.04) | 0.82 (0.33) |
| **3856.62** | 1280 | 0.47 (0.25) | 7E-5 (4E-5) | 0.26 (0.10) | 0.41 (0.24) | 0.29 (0.29) | 0.17 (0.19) | 0.12 (0.06) | 0.87 (0.32) |
| **4893.90** | 1280 | 0.46 (0.20) | 7E-5 (5E-5) | 0.29 (0.09) | 0.45 (0.23) | 0.19 (0.07) | 0.07 (0.07) | 0.13 (0.06) | 0.75 (0.29) |
| **6210.17** | 1280 | 0.66 (0.35) | 9E-5 (6E-5) | 0.24 (0.10) | 0.36 (0.15) | 0.09 (0.14) | 0.51 (0.49) | 0.06 (0.09) | 0.56 (0.25) |
| **7880.46** | 2560 | 0.51 (0.14) | 2E-4 (1E-4) | 0.31 (0.14) | 0.46 (0.18) | 0.28 (0.09) | 0.17 (0.11) | 0.19 (0.06) | 0.68 (0.15) |
| **10000.00** | 1280 | 0.54 (0.22) | 1E-4 (1E-4) | 0.26 (0.14) | 0.31 (0.25) | 0.29 (0.14) | 0.65 (1.13) | 0.16 (0.07) | 0.58 (0.24) |

Table S6: Total-order Sobol indices $S_{T_{i}}$ for medium kinetics. Grey values represent the 95% confidence intervals of the $S_{T_{i}}$. The values are rounded.

| **Koc** | **Model runs** | **PEC_max_** | | | | **LP_50_** | | | |
| --- | --- | --- | --- | --- | --- | --- | --- | --- | --- |
|  |  | **Water depth** | **DT50** | **Koc** | **area** | **Water depth** | **DT50** | **Koc** | **area** |
| **10.00** | 1280 | 1.01 (0.16) | 3E-5 (2E-5) | 0.01 (4E-3) | 4E-3 (4E-3) | 0.84 (0.23) | 0.15 (0.17) | 0.01 (0.01) | 0.01 (0.01) |
| **12.69** | 1280 | 0.91 (0.19) | 1E-4 (1E-4) | 0.08 (0.04) | 0.07 (0.03) | 0.76 (0.20) | 0.07 (0.05) | 0.10 (0.06) | 0.06 (0.03) |
| **16.10** | 1280 | 0.71 (0.29) | 2E-4 (1E-4) | 0.45 (0.20) | 0.25 (0.17) | 0.67 (0.16) | 0.09 (0.08) | 0.36 (0.14) | 0.17 (0.09) |
| **20.43** | 1280 | 0.57 (0.26) | 2E-4 (2E-4) | 0.44 (0.24) | 0.39 (0.20) | 0.47 (0.17) | 0.12 (0.13) | 0.41 (0.12) | 0.33 (0.22) |
| **25.93** | 1280 | 0.48 (0.18) | 9E-5 (7E-5) | 0.42 (0.24) | 0.34 (0.19) | 0.34 (0.12) | 0.22 (0.19) | 0.44 (0.12) | 0.29 (0.16) |
| **32.90** | 1280 | 0.46 (0.17) | 1E-4 (8E-5) | 0.60 (0.31) | 0.43 (0.23) | 0.23 (0.14) | 0.04 (0.04) | 0.35 (0.19) | 0.36 (0.26) |
| **41.75** | 1280 | 0.49 (0.16) | 4E-4 (5E-4) | 0.45 (0.28) | 0.25 (0.13) | 0.26 (0.16) | 0.04 (0.05) | 0.40 (0.16) | 0.47 (0.21) |
| **52.98** | 1280 | 0.38 (0.14) | 1E-4 (2E-4) | 0.52 (0.21) | 0.46 (0.36) | 0.21 (0.12) | 0.05 (0.06) | 0.22 (0.16) | 0.39 (0.27) |
| **67.23** | 1280 | 0.51 (0.37) | 1E-5 (8E-6) | 0.43 (0.24) | 0.58 (0.41) | 0.25 (0.22) | 0.04 (0.05) | 0.25 (0.16) | 0.66 (0.31) |
| **85.32** | 1280 | 0.56 (0.22) | 3E-5 (3E-5) | 0.34 (0.16) | 0.53 (0.29) | 0.20 (0.13) | 0.01 (0.01) | 0.32 (0.16) | 0.95 (0.50) |
| **108.26** | 2560 | 0.58 (0.15) | 5E-4 (1E-3) | 0.19 (0.07) | 0.41 (0.13) | 0.12 (0.10) | 0.03 (0.04) | 0.09 (0.06) | 0.76 (0.24) |
| **137.38** | 1280 | 0.62 (0.19) | 7 E-5 (1E-4) | 0.11 (0.06) | 0.44  (0.17) | 0.09 (0.07) | 0.08 (0.17) | 0.12 (0.11) | 0.82 (0.22) |
| **174.33** | 1280 | 0.60 (0.19) | 5E-5 (6E-5) | 0.05 (0.03) | 0.58 (0.23) | 0.04 (0.07) | 0.24 (0.27) | 0.01 (0.02) | 0.87 (0.24) |
| **221.22** | 1280 | 0.60 (0.19) | 2E-5 (2E-5) | 0.02 (0.01) | 0.43 (0.20) | 0.12 (0.11) | 0.01 (0.01) | 0.01 (0.01) | 0.96 (0.42) |
| **280.72** | 1280 | 0.63 (0.19) | 4E-5 (5E-5) | 0.01 (0.01) | 0.45 (0.18) | 0.11 (0.09) | 0.02 (0.02) | 5E-3 (5E-3) | 0.82 (0.22) |
| **356.22** | 2560 | 0.60 (0.13) | 4E-5 (4E-5) | 0.03 (0.01) | 0.49 (0.16) | 0.29 (0.26) | 0.10 (0.17) | 4E-3 (3E-3) | 0.90 (0.30) |
| **452.04** | 2560 | 0.57 (0.11) | 1E-4 (1E-4) | 0.05 (0.02) | 0.52 (0.24) | 0.20 (0.16) | 0.05 (0.07) | 0.01 (0.01) | 0.80 (0.38) |
| **573.62** | 1280 | 0.60 (0.22) | 5E-3 (1E-3) | 0.09 (0.06) | 0.40 (0.16) | 0.17 (0.15) | 0.04 (0.05) | 0.06 (0.04) | 0.91 (0.43) |
| **727.90** | 1280 | 0.62 (0.18) | 1E-3 (1E-3) | 0.16 (0.08) | 0.49 (0.20) | 0.17 (0.14) | 0.02 (0.02) | 0.02 (0.02) | 0.81 (0.45) |
| **923.67** | 1280 | 0.65 (0.31) | 3E-5 (1E-5) | 0.16 (0.07) | 0.50 (0.19) | 0.10 (0.09) | 0.04 (0.07) | 0.03 (0.03) | 0.96 (0.36) |
| **1172.10** | 1280 | 0.54 (0.26) | 7E-5 (8E-5) | 0.18 (0.10) | 0.37 (0.21) | 0.11 (0.10) | 0.06 (0.06) | 0.09 (0.07) | 0.88 (0.39) |
| **1487.35** | 1280 | 0.57 (0.20) | 8E-5 (8E-5) | 0.20 (0.08) | 0.46 (0.17) | 0.20 (0.13) | 0.03 (0.04) | 0.08 (0.05) | 0.88 (0.31) |
| **1887.39** | 1280 | 0.54 (0.24) | 1E-4 (2E-4) | 0.21 (0.11) | 0.43 (0.22) | 0.13 (0.09) | 0.04 (0.07) | 0.10 (0.07) | 0.62 (0.30) |
| **2395.03** | 2560 | 0.53 (0.17) | 6E-5 (3E-5) | 0.25 (0.09) | 0.45 (0.17) | 0.15 (0.10) | 0.03 (0.03) | 0.06 (0.03) | 0.90 (0.48) |
| **3039.20** | 1280 | 0.61 (0.28) | 5E-5 (3E-5) | 0.27 (0.10) | 0.48 (0.19) | 0.23 (0.12) | 0.03 (0.03) | 0.07 (0.05) | 0.87 (0.34) |
| **3856.62** | 1280 | 0.49 (0.22) | 1E-4 (9E-5) | 0.22 (0.13) | 0.44 (0.24) | 0.36 (0.23) | 0.03 (0.03) | 0.09 (0.07) | 0.87 (0.47) |
| **4893.90** | 1280 | 0.45 (0.16) | 1E-4 (9E-5) | 0.26 (0.16) | 0.42 (0.27) | 0.21 (0.12) | 0.03 (0.05) | 0.11 (0.06) | 0.68 (0.34) |
| **6210.17** | 1280 | 0.52 (0.31) | 9E-5 (6E-5) | 0.24 (0.16) | 0.40 (0.20) | 0.35 (0.22) | 0.03 (0.02) | 0.15 (0.06) | 0.85 (0.40) |
| **7880.46** | 1280 | 0.50 (0.20) | 1E-4 (8E-5) | 0.23 (0.10) | 0.39 (0.20) | 0.23 (0.07) | 0.08 (0.13) | 0.17 (0.10) | 0.64 (0.20) |
| **10000.00** | 1280 | 0.40 (0.13) | 7E-4 (1E-3) | 0.27 (0.12) | 0.33 (0.14) | 0.45 (0.14) | 0.06 (0.05) | 0.12 (0.05) | 0.65 (0.18) |

Table S7: Total-order Sobol indices $S_{T_{i}}$ for fast kinetics. Grey values represent the 95% confidence intervals of the $S_{T_{i}}$. The values are rounded.

| **Koc** | **Model runs** | **PECmax** | | | | **LP50** | | | |
| --- | --- | --- | --- | --- | --- | --- | --- | --- | --- |
|  |  | **Water depth** | **DT50** | **Koc** | **area** | **Water depth** | **DT50** | **Koc** | **area** |
| **10.00** | 1280 | 1.01 (0.17) | 3E-5 (4E-5) | 0.01 (5E-3) | 0.01 (0.01) | 0.96 (0.19) | 0.08 (0.10) | 0.02 (0.02) | 0.02 (0.02) |
| **12.69** | 1280 | 0.95 (0.12) | 3E-4 (3E-4) | 0.09 (0.05) | 0.08 (0.04) | 0.88 (0.17) | 0.03 (0.02) | 0.12 (0.06) | 0.08 (0.06) |
| **16.10** | 1280 | 0.69 (0.22) | 3E-4 (3E-4) | 0.37 (0.20) | 0.18 (0.12) | 0.67 (0.17) | 0.03 (0.02) | 0.31 (0.12) | 0.14 (0.06) |
| **20.43** | 1280 | 0.58 (0.30) | 3E-4 (2E-4) | 0.45 (0.23) | 0.24 (0.15) | 0.52 (0.14) | 0.02 (0.01) | 0.44 (0.15) | 0.31 (0.14) |
| **25.93** | 1280 | 0.37 (0.20) | 1E-4 (8E-5) | 0.43 (0.21) | 0.35 (0.19) | 0.38 (0.14) | 0.02 (0.01) | 0.51 (0.19) | 0.28 (0.10) |
| **32.90** | 1280 | 0.44 (0.17) | 7E-5 (5E-5) | 0.56 (0.25) | 0.35 (0.20) | 0.36 (0.15) | 0.02 (0.01) | 0.51 (0.18) | 0.40 (0.17) |
| **41.75** | 1280 | 0.35 (0.18) | 3E-5 (2E-5) | 0.46 (0.26) | 0.36 (0.18) | 0.25 (0.15) | 0.01 (0.02) | 0.44 (0.21) | 0.39 (0.21) |
| **52.98** | 1280 | 0.47 (0.21) | 3E-5 (2E-5) | 0.37 (0.18) | 0.41 (0.25) | 0.20 (0.13) | 0.01 (0.01) | 0.39 (0.17) | 0.71 (0.33) |
| **67.23** | 1280 | 0.56 (0.24) | 3E-5 (2E-5) | 0.38 (0.17) | 0.48 (0.30) | 0.31 (0.21) | 0.04 (0.05) | 0.37 (0.25) | 0.74 (0.29) |
| **85.32** | 1280 | 0.45 (0.16) | 2E-5 (1E-5) | 0.21 (0.11) | 0.41 (0.19) | 0.35 (0.26) | 0.03 (0.03) | 0.15 (0.10) | 0.75 (0.45) |
| **108.26** | 1280 | 0.54 (0.18) | 3E-5 (3E-5) | 0.18 (0.08) | 0.42 (0.18) | 0.15 (0.14) | 0.01 (0.02) | 0.25 (0.14) | 1.19 (0.74) |
| **137.38** | 1280 | 0.56 (0.18) | 2E-5 (1E-5) | 0.11 (0.09) | 0.45 (0.20) | 0.10 (0.08) | 0.03 (0.04) | 0.11 (0.06) | 1.14 (0.56) |
| **174.33** | 1280 | 0.67 (0.21) | 5E-5 (6E-5) | 0.06 (0.03) | 0.41 (0.15) | 0.13 (0.08) | 0.02 (0.02) | 0.04 (0.03) | 1.10 (0.37) |
| **221.22** | 1280 | 0.76 (0.18) | 1E-3 (2E-3) | 0.01 (0.01) | 0.54 (0.18) | 0.17 (0.16) | 0.02 (0.02) | 0.02 (0.01) | 0.83 (0.18) |
| **280.72** | 1280 | 0.57 (0.17) | 7E-5 (1E-4) | 0.01 (3E-3) | 0.52 (0.24) | 0.09 (0.07) | 0.02 (0.02) | 3E-3 (3E-3) | 0.79 (0.51) |
| **356.22** | 1280 | 0.62 (0.20) | 2E-4 3(E-4) | 0.03 (0.01) | 0.53 (0.20) | 0.15 (0.14) | 4E-3 (5E-3) | 0.01 (8E-3) | 0.96 (0.27) |
| **452.04** | 1280 | 0.59 (0.23) | 4E-5 (4E-5) | 0.05 (0.02) | 0.41 (0.16) | 0.11 (0.08) | 0.03 (0.04) | 0.01 (0.01) | 1.10 (0.28) |
| **573.62** | 1280 | 0.54 (0.24) | 3E-5 (4E-5) | 0.08 (0.04) | 0.53 (0.26) | 0.21 (0.14) | 0.01 (0.02) | 0.05 (0.04) | 0.97 (0.11) |
| **727.90** | 1280 | 0.66 (0.26) | 6E-5 (6E-5) | 0.12 (0.05) | 0.52 (0.24) | 0.13 (0.08) | 0.01 (0.01) | 0.02 (0.01) | 0.96 (0.46) |
| **923.67** | 1280 | 0.54 (0.16) | 9E-5 (1E-4) | 0.14 (0.06) | 0.43 (0.24) | 0.14 (0.08) | 0.02 (0.02) | 0.10 (0.05) | 1.07 (0.33) |
| **1172.10** | 1280 | 0.55 (0.32) | 4E-5 (3E-5) | 0.14 (0.08) | 0.37 (0.17) | 0.14 (0.09) | 0.01 (0.01) | 0.04 (0.03) | 0.98 (0.47) |
| **1487.35** | 1280 | 0.46 (0.17) | 8E-5 (6E-5) | 21 (0.09) | 0.50 (0.30) | 0.27 (0.24) | 0.01 (0.01) | 0.03 (0.03) | 0.77 (0.42) |
| **1887.39** | 1280 | 0.54 (0.22) | 4E-5 (3E-5) | 0.25 (0.10) | 0.48 (0.21) | 0.12 (0.09) | 0.01 (0.01) | 0.05 (0.04) | 0.53 (0.36) |
| **2395.03** | 1280 | 0.55 (0.21) | 1E-4 (1E-4) | 0.27 (0.08) | 0.53 (0.23) | 0.36 (0.24) | 0.04 (0.04) | 0.09 (0.05) | 0.86 (0.39) |
| **3039.20** | 1280 | 0.57 (0.27) | 6E-5 (4E-5) | 0.30 (0.20) | 0.45 (0.18) | 0.10 (0.06) | 0.03 (0.03) | 0.07 (0.05) | 0.85 (0.32) |
| **3856.62** | 1280 | 0.39 (0.27) | 5E-5 (4E-5) | 0.21 (0.13) | 0.36 (0.24) | 0.28 (0.15) | 0.04 (0.04) | 0.10 (0.05) | 0.80 (0.25) |
| **4893.90** | 1280 | 0.51 (0.20) | 1E-4 (9E-5) | 0.28 (0.20) | 0.42 (0.19) | 0.20 (0.10) | 0.01 (0.01) | 0.13 (0.08) | 0.71 (0.24) |
| **6210.17** | 1280 | 0.54 (0.20) | 2E-4 (3E-4) | 0.30 (0.20) | 0.42 (0.22) | 0.30 (0.13) | 0.01 (0.01) | 0.13 (0.07) | 0.71 (0.18) |
| **7880.46** | 1280 | 0.62 (0.40) | 2E-4 (3E-4) | 0.25 (0.11) | 0.35 (0.18) | 0.36 (0.12) | 0.06 (0.07) | 0.16 (0.06) | 0.68 (0.20) |
| **10000.00** | 1280 | 0.52 (0.27) | 3E-4 (4E-4) | 0.35 (0.14) | 0.29 (0.15) | 0.41 (0.13) | 0.01 (0.01) | 0.16 (0.07) | 0.58 (0.16) |

## Visual representation of total-order sobol indices for PEC_max_

Different kinetics do not influence PECmax, as these changes do only affect GUTS dynamics. Nevertheless, comparing PECmax results from the different analyses can be considered as form of validation.

**Slow kinetics**

Figure S3: Total-order Sobol indices for PEC_max_ considering slow kinetics in GUTS.

**Fast kinetics**

Figure S4: Total-order Sobol indices for PEC_max_ considering slow kinetics in GUTS.

## Visual representation of total-order sobol indices for LP_50_

**Slow kinetics**

Figure S5: Total-order Sobol indices for LP_50_ considering slow kinetics.

**Fast kinetics**

Figure S6: Total-order Sobol indices for LP_50_ considering fast kinetics.

1. [https://doi.org/10.2903/j.efsa.2018.5377](https://doi.org/10.2903%2Fj.efsa.2018.5377)
2. https://gitlab.uni-osnabrueck.de/memuos/mempyguts
